# Prevalence of and Risk Factors for Depression Among Older Persons 6 Months After the Lushan Earthquake in China: A Cross-Sectional Survey

**DOI:** 10.3389/fpsyt.2020.00853

**Published:** 2020-09-25

**Authors:** Lan Li, Jan D. Reinhardt, Andrew Pennycott, Ying Li, Qian Chen

**Affiliations:** ^1^ West China School of Nursing/West China Hospital, Sichuan University, Chengdu, China; ^2^ School of Nursing, Southwest Medical University, Luzhou, China; ^3^ The Hong Kong Polytechnic University Institute for Disaster Management and Reconstruction, Sichuan University, Chengdu, China; ^4^ Swiss Paraplegic Research, Nottwil, Switzerland; ^5^ Department of Health Sciences and Health Policy, University of Lucerne, Lucerne, Switzerland; ^6^ Sensory-Motor Systems Lab, Department of Health Science and Technology ETH Zürich, Zürich, Switzerland; ^7^ The Center of Gerontology and Geriatrics, West China Hospital, Sichuan University, Chengdu, China; ^8^ National Clinical Research Center of Geriatrics, West China Hospital, Sichuan University, Chengdu, China

**Keywords:** geriatric depression, earthquakes, social support, activities of daily living, loneliness

## Abstract

**Background:**

Older persons are particularly vulnerable to the impact of earthquakes and are more likely to suffer from depression.

**Objectives:**

We aimed to estimate the prevalence of depression, to compare the prevalence between disaster-affected and non-disaster affected areas, and to explore additional risk factors for depression 6 months after the Lushan earthquake.

**Design:**

A cross-sectional study was conducted.

**Setting:**

A magnitude 7.0 earthquake occurred in Lushan County, Ya’an Prefecture, Sichuan Province, on April 20, 2013. In total, 196 people were killed, and 11,470 were injured over 5 days.

**Participants:**

A multistage cluster sampling strategy was used. A total of 1,509 older persons (aged 60 or older) participated.

**Methods:**

The Geriatric Depression Scale-15, the University of California Los Angeles Loneliness scale, the Activities of Daily Living Scale, the Social Support Rating Scale, and the Family Apgar Index scale were used to evaluate symptoms of depression, loneliness, dependence with respect to activities of daily living, perceived social support, and family function, respectively. A generalized linear regression model and multiple logistic regression analysis were employed to identify risk factors for depression.

**Results:**

Using a cutoff score of 8, the prevalence of depression was 29.16% in the hardest-hit disaster area, 29.06% in the hard-hit disaster area, 31.61% in the moderately-hit disaster area, 17.94% in the remote non-disaster area, and 12.28% in the neighboring non-disaster area. The prevalence was significantly higher in the disaster areas than in the non-disaster areas. Risk factors for depression included an educational level of elementary school or lower, a greater number of chronic illnesses, feelings of loneliness, limitations in activities of daily living, family dysfunction, and low levels of social support.

**Conclusion:**

Depression was highly prevalent in elderly survivors and was significantly more prevalent in disaster areas than in non-disaster areas. Psychological interventions and care should focus on older survivors.

## Introduction

The level of earthquake activity in Sichuan Province ranks fifth in China after Taiwan, Tibet, Xinjiang and Yunnan Province (1). After the 2008 Wenchuan earthquake that occurred in Sichuan in May 2008, a magnitude 7.0 earthquake occurred in Lushan County, Ya’an Prefecture, Sichuan Province, on April 20, 2013, according to the Chinese Seismic Network. In total, 196 people were killed, and 11,470 were injured as of April 25, 2013 (2).

In addition to causing injury and death, earthquakes have a negative influence on the mental health of survivors (3). Depression is one of the most common mental health problems following such a disaster (4). Most older persons suffer from a decline in physical and mental health, social segregation, poor economic conditions, poor access to resources, communication difficulties, and a lack of access to information. These experiences may increase levels of pessimism, loneliness, sleep disturbance, and depression.

Indeed, these problems are even more serious after a destructive earthquake. Accordingly, older persons are particularly vulnerable to the adverse effects of earthquakes (5), and an unexpected destructive earthquake can overwhelm elderly people (6).

Geriatric depression (GD) is a critical public health problem since it is the leading cause of disability and the fourth leading cause of disease burden (Ustun and Ayuso‐Mateos, 2004). It is associated with increased morbidity and mortality, including suicide, as well as decreased physical, cognitive, and social functioning (7, 8). Non-suicidal mortality is one of the significant adverse results of GD, which has been shown to be positively correlated with mortality in 72% of elderly adults (9). GD was shown to increase disability and mobility limitations over 6 years by 67% and 73%, respectively (10), and being single, living alone, poor sleep quality, and stress in daily life—due to financial and other concerns—are common reasons that elderly people with depression commit suicide (11).

Some studies have been conducted on the prevalence of and risk factors for depression among elderly survivors after the 2008 Wenchuan earthquake, the Great East Japan earthquake in 2011 and the Lushan earthquake of 2013. A study by Xie et al. analyzed risk factors for depression in 189 elderly persons (aged 65 years or older) from the hardest-hit disaster area of Mianzhu living in temporary houses 13 months after the Wenchuan earthquake (12). They found that reduced independence with respect to instrumental activities of daily living (IADL) was associated with a greater probability of depression. According to a recent study (13), 83.1% of 1783 older solitary people (aged 60–75 years) from the most severely affected disaster area (Dujiangyan, Pengzhou, Shifang, etc.) showed symptoms of depression seven years after the Wenchuan earthquake as measured by the GD Scale-30 (GDS-30). The major risk factors for depression were being female, being single, bereavement, poor perceived level of social support, and insufficient support availability. In addition, 37.2% of 163 elderly survivors (aged 65 years or older) displayed depressive symptoms two years after the Great East Japan earthquake as measured by the Basic Checklist (BCL). The likelihood of depression was significantly increased for females, those of older age, people with a history of diabetes, and people with a history of cognitive disorders. Elderly survivors with a higher level of independence with respect to IADL and engagement in social activities had, in turn, a reduced prevalence of depression (14). Another study on 3,464 older survivors (aged 65 years or older) conducted 3 years after the Great East Japan earthquake reported that loss of property and employment and not obtaining psychiatric care were signiﬁcantly related to depressive symptoms (15).

Only one previous study has focused on depression in elderly survivors of the Lushan earthquake. Liang and colleagues (16) used the GDS-30 to assess risk factors for depression among 691 elderly persons (aged 60 years and above) from the hard-hit disaster area (Ya’an) at an unknown time point after the Lushan earthquake. They found that higher age, being single and female, having a lower education level, low income, and anxiety were risk factors for depression. However, they did not report the prevalence of depression.

Previous research has demonstrated that GD is prevalent among elderly earthquake survivors and that risk factors for GD include sociodemographics, earthquake-related experiences, mental and physical health, and resource shortages. However, it is unclear how the prevalence of GD varies by the effect of the disaster on the area where people live. In the Lushan earthquake, disaster-affected areas were classified as follows: 1) hardest-hit area (Lushan County), 2) hard-hit area (Yucheng District, Tianquan County, Yingshan District, Yingjing County, and Baoxing County), 3) moderately-hit area (Shimian County, Ganluo County and Yanyuan County), 4) remote non-disaster area (Nanchong Prefecture), and 5) neighboring non-disaster area (Chengdu Prefecture) (17). The purpose of the present study was to 1) estimate the prevalence of depression in disaster-affected areas and non-disaster after the Lushan earthquake, 2) compare the prevalence of depression in disaster and non-disaster areas, and 3) identify other risk factors for depression in elderly Lushan earthquake survivors, including comorbidities.

## Methods

### Participants and Procedure

We analyzed data from a cross-sectional study conducted approximately six months (from October 20 to November 10, 2013) after the Lushan earthquake.

Multistage stratified random sampling was combined with snowball sampling in this study. First, according to the assessment report on the “April 20” strong earthquake disaster in Lushan, Sichuan, the study sites were divided into five different areas (17). Second, one county (Lushan) was selected from the hardest-hit disaster area, and two counties were selected at random from each of the other four areas. Thus, nine counties were selected in total. Third, four to five communities were randomly selected in each of the five different areas. In these communities, elderly persons were approached at central locations. Participants were encouraged to recommend other elderly persons living in their community to participate in the study, thus employing a snowball sampling method.

Inclusion criteria were as follows: aged 60 years and older, residing in one of the study areas during the Lushan earthquake, and having provided informed consent. People were excluded if serious communication difficulties occurred during the interview.

The survey was carried out by investigators who had been trained concerning the purpose of the survey, in the use of the scales, regarding the content of the questionnaire, and communication skills. Older persons who could read and write completed the questionnaire by themselves. Face-to-face interviews were conducted with illiterate persons and those with severely impaired eyesight.

### Measures

#### Main Outcome: GDS-15

The Geriatric Depression Scale-15 (GDS-15) is an abbreviated version of the standardized GDS-30 by Sheikh and Yesavage (18) that has been specifically designed for older persons. A Chinese version of the GDS-15 has been validated, and the scale has demonstrated good internal consistency reliability with a Cronbach’s alpha of 0.84 (19). Indeed, in this survey, the Cronbach’s alpha was found to be 0.889.

The only Chinese study formally establishing a cutoff based on sensitivity and specificity was conducted in a population of 193 elderly residents of Hong Kong. The authors recommended a threshold of 8, yielding a sensitivity of 0.96 and a specificity of 0.88 (20). However, in other studies in mainland China, cutoff scores of 5 and 6 were employed for community-dwelling elderly persons or elderly inpatients (see **Table 1**).

**Table 1:** Prevalence of depression in older adults by the mean of GDS-15 score used in different studies in mainland China.

Study	Population	Sample	Cutoff score	Prevalence	Risk factors
Our study	Elderly people in disaster area	962	5	62.27%	Education level of elementary school or lower, chronic illnesses, feelings of loneliness, limitations in activities of daily living, family dysfunction and low levels of social support
			6	51.35%	
			8	26.74%	
	Elderly people in non-disaster area	547	5	43.14%	_
			6	31.44%	
			8	12%	
Ho et al. (21)	Elderly people in the community	2,604	5	13.3%	Higher body mass index
Wang et al. (22)	Elderly people in the community	948	5	8.2%	Sarcopenia status
Qiu et al. (23)	Elderly patients in a hospital	210	5	17.4%	Total blood protein and hemoglobin levels
She et al. (24)	Elderly people in the community	1,497	5	26.6%	Multimorbidity
Liang et al. (25)	Elderly people in long-term care facilities	595	6	8.7%	Passive suicidal ideation, sleep disturbance, sad, painful, worried facial expressions
Ina et al. (26)	Elderly postmenopausal women in the community	336	6	29.2%	Not reported
Zhi, et al. (27)	Elderly people in the community	1,732	6	9.82%	Lower body mass index, waist circumference and waist-hip ratio
Liu et al. (28)	Elderly people in the community	1,749	6	9.9%	A moderate decline of estimated glomerular filtration rate
Yao et al. (29)	Elderly people in the community	940	6	32.2%	Vitamin D deficiency
Lu et al. (30)	Hospitalized elderly patients with hypertension	457	6	18.38%	Not reported
Pan et al. (31)	Elderly people with mild cognitive impairment in the community	157	8	29.9%	Being single, cognitive decline, decrease of ADL
Niu et al. (32)	Elderly people in the community	484	12	Unclear	Not reported

In the present study, we thus provide prevalence rates for cutoff scores of 5, 6, and 8 to assure optimal comparability with previous research (with one exception, see **Table 1**). For the analysis of risk factors for depression, the most conservative and formally established cutoff score of 8 was used.

#### Exposures

##### Loneliness Scale

The University of California Los Angeles (UCLA) Loneliness Scale was designed by Russell in 1978 to evaluate loneliness arising from the disparity between the desire for social interaction and the actual level of loneliness (33). Wang revised the third edition of the scale in 1995, and it has been widely used to measure loneliness in various groups of people in China (34). The split-half reliability has been reported to be 0.930 (34). The scale is one-dimensional and consists of 20 items. There are 11 positive and 9 negative items, and each item has four response options, with 1 for “never,” 2 for “rarely,” 3 for “sometimes,” and 4 for “all the time.” The overall score ranges from 20 to 80; higher scores indicate more serious symptoms of loneliness. In this survey, the Cronbach’s alpha was 0.775.

##### ADL Scale

The ADL scale was developed by Lawton and Brody (35). The scale has 14 items across two subscales: the Physical Self-Maintenance Subscale (PSMS) and IADL. The PSMS has six items, and the IADL has eight items. Each item has four response options, with 1 for “independent,” 2 for “partially independent,” 3 for “requiring substantial help,” and 4 for “completely dependent.” The total score ranges from 14 to 56, with greater scores indicating a greater degree of dependence. The previously reported Cronbach’s alpha value was 0.768 (12), while in this survey, the Cronbach’s alpha for the ADL Scale was 0.855.

##### Social Support Rating Scale (SSRS)

The SSRS was designed by Xiao et al. (36) in 1944 and has been widely used in the Chinese general population. It comprises 10 items across three dimensions, objective support, subjective support, and utilization of social support. A higher score indicates a higher level of social support. The total score ranges from 0 to 66 and is calculated as the sum of the scores across the three dimensions. The internal consistency as measured by Cronach’s alpha has ranged from 0.89 to 0.94 in previous studies (36). In this survey, Cronach’s alpha for the SSRS was 0.811.

##### Family Apgar Index Scale (APGAR)

The APGAR scale was developed by Smilkstein (37) to evaluate the adaptation, partnership, growth, affection, and resolve (APGAR) of family members (37). It contains five items, and there are three response options for each item: 2 for “often,” 1 for “sometimes,” and 0 for “hardly ever.” The total score ranges from 0 to 10 and is calculated as the sum of the five items. Higher scores indicate poorer function in daily living. The Cronbach’s alpha was previously reported to be 0.787 (12). In this study, Cronbach’s alpha for the APGAR was 0.842.

### Other Variables

#### Sociodemographic Factors

The influence of sociodemographic factors as risk factors for depression were also explored in this research including age, gender, educational level, and marital status. The age of participants was divided into two groups (60–74 years and older than 75 years), while the educational level was composed of elementary school or lower, middle school, and senior school or higher. In addition, the influence of being single or married, and gender was also investigated.

#### Number of Chronic Illnesses Reported

As China becomes an aging society, great changes have taken place in the disease spectrum of Chinese residents. Chronic diseases have become prevalent in the elderly. The incidence and prevalence of chronic diseases (e.g., cardiovascular and cerebrovascular diseases, metabolism, and respiratory system diseases) are on the rise (38). The following conditions were included: a) hypertension, b) diabetes, c) coronary disease, d) chronic obstructive pulmonary disease, and e) cancer. Based on the chronic illnesses reported, we created a sum index for the number of chronic illnesses for further analysis ranging from 0 to 5.

#### Earthquake-Related Variables

The variable assessing the residence’s distance from the epicenter of the earthquake included moderately-hit disaster areas, neighboring non-disaster areas such as Chengdu, and remote non-disaster areas such as Nanchong. Additional variables included the presence of house damage, having been injured, and having family members who were injured or had died.

### Statistical Analysis

Prevalence rates for the different study areas and the difference in rates between the disaster and non-disaster areas were calculated for different cutoff scores of the GDS-15 (5, 6, and 8). Confidence intervals for the prevalence rates were derived from logistic regression.

An analysis of additional risk factors for depression in earthquake victims was confined to the subsample living in the disaster affected areas (n = 962). Univariate and multivariate analyses on the prevalence of depression across potential risk factors were based on logistic regression, yielding unadjusted and adjusted odds ratios, respectively, with 95% confidence intervals.

Univariate analysis of the severity of depressive symptoms provided medians and lower and upper quartile values of GDS-15 scores for categorical independent variables. The statistical tests were the Mann-Whitney U test (binary independent variables) and Kruskal-Wallis equality-of-populations rank test (categorical variables with more than two categories). Spearman correlation coefficients were used to examine the association of GDS-15 scores with continuous independent variables. Multivariable analysis of symptom severity (GDS-15 scores) employed a generalized linear regression model from the negative binomial family with a logit link. This model was most suited to the distribution of the outcome and showed a superior fit compared to ordinary least squares regression.

The independent variables in all the multivariate models were the geographical area (hardest-hit, hard-hit, moderately-hit), age, gender, marital status (married vs. single), educational level (elementary school or lower, middle school, senior school, or higher), house damage, injury of the responders themselves, injury or death of a family member, the number of reported chronic illnesses, social support (SSRS total score), family function (APGAR scale total score), loneliness (UCLA scale total score), and limitations in ADL (ADL scale total score).

Two-sided testing was used in all tests, and the significance level was set at 0.05. Stata 14.0 for Windows (StataCorp LLC, College Station, Texas, U.S.) was used for all analysis.

## Results

A total of 1,600 questionnaires were distributed and 1,509 valid questionnaires were returned including 962 participants from the disaster area and 547 from the non-disaster area. The mean age of the study participants was 71.50 years. Sample descriptive statistics are shown in **Table 2**.

**Table 2:** Descriptive characteristics of the 1,509 participants.

Variables	n(％)	Prevalence of depression n(％)		
		Cutoff of 5	Cutoff of 6	Cutoff of 8
Area				
Hardest-hit disaster area	535(35.45)	332(62.06)	271(50.65)	156(29.16)
Hard-hit disaster area	234(15.51)	141(60.26)	117(50.00)	68(29.06)
Moderately-hit disaster area	193(12.79)	126(65.28)	106(54.92)	61(31.61)
Remote non-disaster area: Nanchong	262(17.36)	128(48.85)	88(33.59)	47(17.94)
Neighboring non-disaster area: Chengdu	285(18.89)	108(37.89)	84(29.47)	35(12.28)
Age(years)				
60–74	991(65.67)	540(54.49)	425(42.89)	233(23.51
≥75	518(34.33)	295(56.95	241(46.53)	134(25.87)
Gender				
Female	701(46.45)	391(55.78)	319(45.51)	176(25.11)
Male	808(53.55)	444(54.95)	347(42.95)	191(23.64)
Marital status				
Single	446(29.56)	281(63.00)	240(53.81)	126(28.25)
Married	1,063(70.44)	554(52.12)	426(40.08)	241(22.67)
Education level				
Elementary school or lower	844(55.93)	511(60.55)	417(49.41)	256(30.33)
Middle school	334(22.13)	175(52.40)	137(41.02)	65(19.46)
Senior school or higher	331(21.94)	149(45.02)	112(33.84)	46(13.90)
House damage due to the earthquake				
No	511(33.86)	229(44.81)	174(34.05)	89(17.42)
Yes	998(66.14)	606(60.72)	492(49.30)	278(27.86)
Suffered injuries due to the earthquake				
No	1,414(93.7)	783(55.37)	630(44.55)	349(26.68)
Yes	95(6.3)	52(54.74)	36(37.89)	18(18.95)
Family members suffered injuries due to the earthquake				
No	1,376(91.19)	762(55.38)	596(43.31)	331(24.06)
Yes	133(8.81)	73(54.89)	70(52.63)	36(27.07)
Number of chronic illnesses reported				
0	662(43.87)	319(48.19)	243(36.71)	125(18.88)
1	522(34.59)	293(56.13)	242(46.36)	130(24.90)
2	229(15.18)	154(67.25)	123(53.71)	73(31.88)
3	66(4.37)	46(69.70)	40(60.61)	28(39.40)
4	30(1.99)	23(76.67)	18(60.00)	11(36.67)
Social support (SSRS total score, median = 34)				
<34	697(46.19)	433(62.12)	360(51.65)	223(31.99)
≥34	812(53.81)	402(49.51)	306(37.68)	144(17.73)
Family function (APGAR scale total score, median = 5)				
<5	211(13.98)	169(80.09)	147(69.67)	93(44.08)
≥5	1298(86.02)	666(51.31)	519(39.98)	274(21.11)
Loneliness (UCLA scale total score, median = 43)				
<43	724(47.98)	293(40.47)	221(30.52)	111(15.33)
≥43	785(52.02)	542(69.04)	445(56.69)	256(32.61)
Limitations in ADL (ADL scale total score, median = 16)				
<16	737(48.84)	325(44.10)	247(33.51)	112(15.20)
≥16	772(51.16)	510(66.06)	419(54.27)	255(33.03)

SSRS, social support rating scale; APGAR, the adaptation, partnership, growth, affection, and resolve of family members; UCLA, University of California Los Angeles; ADL, activities of daily living.

### Prevalence of Depression According to the GDS-15

Estimates for the prevalence of depression based on different cutoff criteria for the GDS-15 total score are given in **Table 2**, along with summary statistics of the study participants. Regardless of the chosen cutoff, the prevalence of depression was highest in the moderately-hit disaster areas and lowest in the neighboring non-disaster areas. The prevalence of depression across all the disaster areas was significantly different from that across all the non-disaster areas, i.e., the 95% confidence intervals did not overlap.

Conversely, there were no significant differences between the two non-disaster areas or among the three disaster areas. Estimates for excess morbidity due to probable depressive disorder ranged from approximately 20% (cutoff score of 6 for the GDS-15) to 14.6% (cutoff score of 8 for the GDS-15) (see **Table 3** for detailed estimates and 95% CIs).

**Table 3:** Prevalence of depression in different study areas with 95% confidence intervals accordingly to GDS-15 cutoff.

Variables	Cutoff of 5			Cutoff of 6			Cutoff of 8		
	Estimate	95%*CI*		Estimate	95%*CI*		Estimate	95%*CI*	
By specific disaster area									
Hardest-hit disaster area	0.621	0.579	0.662	0.507	0.464	0.549	0.292	0.253	0.330
Hard-hit disaster area	0.603	0.540	0.665	0.500	0.436	0.564	0.291	0.232	0.349
Moderately-hit disaster area	0.653	0.586	0.720	0.549	0.479	0.619	0.316	0.250	0.382
Remote non-disaster area: Nanchong	0.489	0.428	0.549	0.336	0.279	0.393	0.179	0.133	0.226
Neighboring non-disaster area: Chengdu	0.379	0.323	0.435	0.295	0.242	0.348	0.123	0.085	0.161
By disaster vs. Non-disaster area									
Disaster area	0.623	0.592	0.653	0.514	0.482	0.545	0.296	0.267	0.325
Non-disaster area	0.431	0.390	0.473	0.314	0.276	0.353	0.150	0.120	0.180
Total	0.553	0.528	0.578	0.441	0.416	0.466	0.243	0.222	0.265
Difference	0.191	0.140	0.243	0.199	0.149	0.249	0.146	0.105	0.188

CI, confidence interval.

### Risk Factors for the Prevalence of Probable Depression

Univariate and multivariate analyses of the prevalence of depression by potential risk factors are presented in **Table 4**. The odds of being positively screened for depression were significantly reduced in people who perceived a higher level of social support, in people with better family function, and in people with a middle school educational level compared to those with an elementary school level of education or lower.

**Table 4:** Univariate (unadjusted) and multivariate (adjusted) analyses of the prevalence (cutoff score of 8) of depression by potential risk factors (n = 962).

Variables	Unadjusted				Adjusted			
	*OR*	*P*	95%*CI* for OR		*OR*	*P*	95%*CI* for *OR*	
			Lower	Upper			Lower	Upper
Area (reference: hardest hit disaster area)								
Hard-hit disaster area	0.995	0.978	0.710	1.396	0.954	0.815	0.644	1.413
Moderately-hit disaster area	1.123	0.524	0.786	1.603	1.193	0.457	0.749	1.902
Age (years)	1.028	0.002	1.010	1.046	1.000	0.997	0.978	1.023
Gender (reference: male)								
Female	1.038	0.795	0.786	1.370	1.013	0.934	0.742	1.384
Marital status (reference: married)								
Single	1.287	0.100	0.953	1.738	0.568	0.004	0.385	0.837
Education level (reference: Elementary school or lower)								
Middle school	0.560	0.002	0.388	0.809	0.585	0.010	0.389	0.882
Senior school or higher	0.671	0.116	0.408	1.104	0.716	0.260	0.400	1.281
House damage due to the earthquake (reference: no)								
Yes	1.240	0.331	0.803	1.915	1.244	0.422	0.729	2.123
Suffered injuries due to the earthquake (reference: no)								
Yes	0.700	0.290	0.362	1.355	0.506	0.086	0.232	1.102
Family members suffered injuries due to the earthquake (reference: no)								
Yes	1.305	0.264	0.818	2.081	1.652	0.075	0.950	2.871
Number of chronic illnesses reported	1.485	<0.001	1.288	1.712	1.258	0.009	1.059	1.495
Social support (SSRS total score)	0.932	<0.001	0.912	0.953	0.954	0.001	0.929	0.981
Family function (APGAR scale total score)	0.831	<0.001	0.782	0.883	0.910	0.012	0.845	0.979
Loneliness (UCLA scale total score)	1.074	<0.001	1.053	1.096	1.065	<0.001	1.041	1.090
Limitations in ADL (ADL scale total score)	1.068	<0.001	1.049	1.088	1.049	<0.001	1.026	1.073
Cos					0.083	0.027	0.009	0.754

OR, odds ratio; CI, confidence interval; SSRS, social support rating scale; APGAR, the adaptation, partnership, growth, affection, and resolve of family members; UCLA, university of California Los Angeles; ADL, activities of daily living.

In turn, the probability of depression was increased in those reporting a greater number of chronic illnesses, participants with higher scores on the UCLA loneliness scale, and people with worse functioning in ADL. Those with older age showed an increased likelihood of depression in the univariate analysis only. Accordingly, the odds of depression were decreased in single persons in the adjusted analysis compared to the unadjusted analysis.

### Association Between the Severity of Depressive Symptoms and Risk Factors

The mean depression score was 5.30 (95% CI: 5.14–5.46) for the total sample, 5.84 in the disaster area (95% CI: 5.64–6.04), and 4.36 (95% CI: 4.11–4.61) in the non-disaster area. A univariate analysis of the severity of depression is presented in **Table 5**.

**Table 5:** Univariate analysis of the association of the severity of depressive symptoms (GDS-15 total score) with potential risk factors (n = 962).

**Categorical variables**	**1st quartile**	**Median**	**2nd quartile**	***P***
Area				
Hardest-hit disaster area	4	6	8	0.846^#^
Hard-hit disaster area	3	5.5	8	
Moderately-hit disaster area	3	6	8	
Gender				
Male	3	6	8	0.636^$^
Female	4	6	8	
Marital status				
Married	3	5	8	<0.001^$^
Single	4	6	9	
Education level				
Elementary school or lower	4	6	8	0.014^#^
Middle school	3	5	7	
Senior school or higher	3	5	7	
House damage due to the earthquake				
No	3	6	8	0.773^$^
Yes	3	6	8	
Suffered injuries due to the earthquake (reference: no)				
No	3	6	8	0.398^$^
Yes	3	5	7	
Family members suffered injuries due to the earthquake (reference: no)				
No	3	6	8	0.607^$^
Yes	4	6	8	
Continuous Variables	r	P		**
Age(years)	0.054	0.035		
Number of chronic illnesses reported	0.223	<0.001		
Social support (SSRS total score)	-0.247	<0.001		
Family function (APGAR scale total score)	-0.213	<0.001		
Loneliness (UCLA scale total score)	0.308	<0.001		
Limitations in ADL (ADL scale total score)	0.308	<0.001		

^#^P-value for chi-squared from Kruskal-Wallis test; ^$^P-value from Z from Mann-Whitney U test.

SSRS, social support rating scale; APGAR, the adaptation, partnership, growth, affection, and resolve of family members; UCLA, University of California Los Angeles; ADL, activities of daily living.

Participants who were single and those with elementary school or lower educational levels presented more depressive symptoms. Age, the number of chronic illnesses reported after earthquake, loneliness, and dependence for ADL were significantly positively correlated with GDS-15 scores. Social support and family function were, in turn, significantly related to decreased symptom severity. The results of the generalized linear model from the negative binomial family are shown in **Table 6**. Respondents with higher scores on the UCLA loneliness scale and people with more limitations in ADL showed significantly increased depressive symptoms.

**Table 6:** Results of the generalized linear model for the association of depressive symptom severity (GDS-15 total score) with potential risk factors (n = 962).

Variables	Coef.	Std. Err.	*Z*	*P*	95% CI	
Area (reference: hardest hit disaster area)						
Hard hit disaster area	-0.071	0.091	-0.780	0.434	-0.250	0.107
Moderately hit disaster area	-0.088	0.113	-0.780	0.433	-0.310	0.133
Age (years)	0.003	0.005	0.560	0.577	-0.008	0.014
Gender (reference male)						
Female	0.004	0.073	0.060	0.956	-0.138	0.146
Marital Status (reference: married)						
Single	-0.033	0.090	-0.370	0.714	-0.209	0.143
Education level (reference: elementary school or lower)						
Middle school	-0.092	0.090	-1.020	0.308	-0.269	0.085
Senior school or higher	-0.073	0.128	-0.570	0.569	-0.323	0.178
House damage due to the earthquake (reference: no)						
Yes	-0.053	0.122	-0.440	0.663	-0.293	0.187
Suffered injuries due to the earthquake (reference: no)						
Yes	-0.103	0.168	-0.610	0.539	-0.432	0.226
Family members suffered injuries due to the earthquake (reference: no)						
Yes	0.053	0.133	0.400	0.689	-0.208	0.314
Number of chronic illnesses reported	0.058	0.042	1.370	0.172	-0.025	0.141
Social support (SSRS total score)	-0.011	0.006	-1.780	0.076	-0.024	0.001
Family function (APGAR scale total score)	-0.017	0.018	-0.980	0.325	-0.052	0.017
Loneliness (UCLA scale total score)	0.019	0.005	3.740	<0.001	0.009	0.028
Limitations in ADL (ADL scale total score)	0.013	0.006	2.330	0.020	0.002	0.024
Cons	1.050	0.514	2.040	0.041	0.042	2.058

OR, odds ratio; CI, confidence interval; SSRS, social support rating scale; APGAR, the adaptation, partnership, growth, affection, and resolve of family members; UCLA, University of California Los Angeles; ADL, activities of daily living.

## Discussion

Depression was found to be highly prevalent in elderly persons six months after the Lushan earthquake. Furthermore, the prevalence of depression was significantly higher in the earthquake-affected areas than in the areas not directly affected by the Lushan earthquake. Loneliness and dependence in ADL were persistent risk factors for probable depression as well as increased symptom severity in elderly Lushan earthquake survivors across all models. In addition, low education, less perceived social support, worse family function, and reporting a greater number of chronic illnesses were persistent risk factors for depression across in unadjusted and adjusted logistic regression models.

The only other study to date on elderly earthquake victims was conducted by Xu and colleagues (13), who administered the GDS-30 to 1,783 elderly people living alone 7 years after the Wenchuan earthquake. They employed a cutoff score of 10, which is comparable with a cutoff score of 5 when using the GDS-15. A prevalence rate of 83.1% was reported, which was higher than the rate reported in our study. However, the authors concentrated on a particularly vulnerable subpopulation.

Regarding the prevalence of depression in elderly citizens from mainland China, the prevalence rates found in our study were higher, irrespective of the GDS-15 cutoff used, with one exception (see **Table 1**). A study by Pan et al. (31) found a prevalence rate of depression of approximately 30% in a population of elderly persons with mild cognitive impairments, which was approximately 3% higher than the prevalence estimated in the disaster area of our study when using a GDS-15 cutoff of eight points.

The prevalence of depression in the non-disaster areas in our study was also higher than the rates reported in most other general population studies. This difference may be related to the indirect effects of the Lushan earthquake such as an increased perception of risk and the effects of the Wenchuan earthquake that affected almost the entire Sichuan region 5 years earlier.

The prevalence of depression was highest in the moderately-hit as opposed to the hardest-hit areas. A potential explanation is that the survivors in the hardest-hit area disaster areas received more disaster relief after the destructive earthquake. Such disaster relief often included psychological, material, and financial support, which, to some extent, alleviated the psychological burden on the survivors. In addition, according to a document issued by the Chinese State Council—a notice concerning the issuance of a plan for post earthquake recovery and reconstruction in Lushan—the counties (e.g. Yucheng district) of the hardest-hit areas were the focus of policy support from the government in post-disaster reconstruction. Therefore, in the hardest-hit disaster areas, the local government had more resources available for improving quality of life of the local residents, including medical, personnel, and financial support, which may have alleviated the effects of the earthquake with regard to depression.

Instead of prevalence rates, some previous studies have provided mean GDS-15 total scores. Xie and colleagues (12) reported a mean score of 5.27 in a study of 189 older persons living in temporary houses 13 months after the Wenchuan earthquake, which is higher than the mean GDS-15 score in the non-disaster affected area of our study but lower than the average GDS-15 score found in the disaster area. Tsuboya and colleagues (15) reported a mean GDS-15 score of 3.8 in a survey of 3567 elderly persons 3 years after the Great East Japan earthquake, which is lower than the average GDS-15 score found in the disaster-affected area and non-disaster affected area of our study. Xie et al. (39) reported a mean score of 4.57 in a study of Chinese patients in hospitals, which is also lower than the scores from disaster areas and non-disaster areas in our study.

We found that elderly people from the disaster area showed a higher prevalence of depression than those from the non-disaster area, indicating excess morbidity due to the disaster. This finding is novel as no previous study in elderly earthquake survivors has employed a geographical control group. The only other study estimating depression-related morbidity assessed 3,464 elderly earthquake survivors and compared baseline mean GDS-15 scores at 11 months before the Great East Japan earthquake with mean scores after the earthquake (15). No significant difference was found (mean before: 3.74, 95% CI: 3.38–3.62 vs. mean after: 3.84, 95% CI: 3.39–3.51).

Elderly people are a group for which various psychological issues such as depression are common even under normal circumstances (40). These problems can be even more pronounced in the wake of a natural disaster; for instance, approximately one-third elderly people surveyed in one study were found to have symptoms of depression (41). Elderly people could be at greater risk of negative impacts due to pre-existing physical disorders, prior bereavement, and limited financial resources, for example, (42). Indeed, some research has found that elderly people are at greater risk of psychological issues following disasters (43, 44).

We also found that areas differently affected by the disaster did not differ significantly in prevalence rates of depression, and that there was no significant difference between the remote and neighboring non-disaster areas; these results have not previously been reported. A possible explanation may be that the Lushan earthquake, which had a magnitude of 7.0, was not much more destructive than the Wenchuan earthquake, and Lushan earthquake survivors could also benefit from previous experience regarding the provision of psychological relief and medical aid gained from the Wenchuan earthquake.

Participants who reported being lonely were more likely to be screened positively for depression and showed an increased severity of depressive symptoms. This finding is consistent with a meta-analysis of the effect of loneliness on depression that included 88 research papers with a total of 40,068 study participants (45) and studies analyzing loneliness among elderly people in particular (46, 47). Negative emotions induced by loneliness have a chronic and recurrent structure and may play a key role in the development and persistence of mental health problems (48, 49). Indeed, the hypothesis that negative emotions play an important role in the development of mental health issues has recently been further validated by neuroimaging studies, pointing out that traumatic events may affect brain structures, specifically those involved in emotion regulation mechanisms (50). Furthermore, negative emotions may directly impact depression severity and clinical outcomes of mood disorders (51).

Another consistent risk factor for increased severity of depressive symptoms found in our study was ADL dependence, confirming previous findings. Depression was, for instance, strongly associated with dysfunction in IADL among 2,695 community-dwelling elderly from three Asian countries: Indonesia, Vietnam, and Japan (52). This association has also been shown in a sample of 763 older people in Japan who were observed over a 7.5 year prospective period (53), in 189 older persons living in temporary houses at 13 months after the Wenchuan earthquake (12), and in 163 older survivors 2 years after the Great East Japan earthquake (14). Decreases in function related to aging or physical trauma and the loss of social roles can induce depression and other negative emotions in elderly people.

A low level of social support was a risk factor for depression among elderly survivors. It was, however, not significantly associated with depressive symptoms in the multivariate analysis on symptom severity, which may be explained by its multicollinearity with loneliness. In line with the present study, elderly individuals with high levels of social support showed a lower prevalence of depression after the 1999 Taiwan earthquake (54). Social support also negatively predicted depression in a study of general population survivors 6 months after the Wenchuan earthquake (55). Participants receiving social support from friends or neighbors had fewer depressive symptoms.

However, those receiving organizational support have shown more depressive symptoms (56). Higher social support means greater opportunity to have contact with families, friends, neighbors, and other people. Moreover, social support means that more resources can be mobilized from households, communities, the surrounding society and governments. Older persons with a higher level of social support also tend to use more effective coping strategies to avoid mental health problems such as depression and feelings of loneliness (57).

Our identification of family dysfunction as another risk factor for depression has not been previously reported for earthquake survivors. Previous studies, however, have reported that elderly people living in nursing homes (58), residing in the community (59, 60), and inpatients (61) with poor family function were more likely to present depressive symptoms. Family function reflects the ability of a family as a whole to meet the needs of each of its members and is closely related to the health of the individual. With increasing age, the physical condition and self-care ability of elderly people gradually declines. In China, the family traditionally takes on the role of carer for its elderly members and provides economic support and spiritual comfort. A disruption in family function can thus give rise to poverty, isolation, and a lack of medical care when needed.

A further risk factor for depression in our study was the number of chronic illnesses. This is in line with previous studies that have demonstrated an increased risk of depression in elderly people with a chronic disease (62) and multimorbidity (24). Older persons with chronic diseases need long-term medication and monitoring, but the management of chronic diseases can become problematic in the wake of a disaster because of possible disruption of the medical supply chain (63). Moreover, chronic diseases may be worsened by stress and changes in living conditions such as having to move to temporary shelters (64).

There are some limitations of the present study that warrant mention. First, the data were collected based on a self-reported approach that may have had biased results. Second, baseline data on depression were lacking, so it cannot be ruled out that differences in prevalence rates between the disaster and non-disaster areas may not be related to the earthquake but rather to other features of the environment not assessed in the study. Third, it should be noted that one of our control areas neighbored the earthquake zone and the other more remote area was similar with regard to socioeconomic conditions.

## Conclusions

The prevalence of depression was high in elderly survivors of the Lushan earthquake and significantly higher than in areas of Sichuan that were not directly affected by this disaster. The risk factors for depression, which include a decline in ADL function, suffering from chronic illnesses, loneliness, a lack of social support, and disrupted family function, are potentially important intervention targets for public health workers with regard to preventing and reducing depression in elderly earthquake survivors.

## Data Availability Statement

The datasets generated for this study are available on request to the corresponding author.

## Ethics Statement

The studies involving human participants were reviewed and approved by The West China Hospital Medical Ethics Committee at Sichuan University (Sichuan, China). The patients/participants provided their written informed consent to participate in this study. Written informed consent was obtained from the individual(s) for the publication of any potentially identifiable images or data included in this article.

## Author Contributions

This study was designed by QC. Data were collected by YL. Data were analyzed and interpreted by LL and JR. Language was polished by AP. LL wrote the first draft. All other authors revised the manuscript for critical content.

## Funding

This work was supported by the Post-Earthquake Reconstruction Research Project of Sichuan University (no. 2013SCU19009) and the Plan of Sichuan Science and Technology Department (2019YFS0390).

## Conflict of Interest

The authors declare that the research was conducted in the absence of any commercial or financial relationships that could be construed as a potential conflict of interest.
